# Structural Insights into the Recognition of Phosphopeptide by the FHA Domain of Kanadaptin

**DOI:** 10.1371/journal.pone.0107309

**Published:** 2014-09-08

**Authors:** Qingping Xu, Marc C. Deller, Tine K. Nielsen, Joanna C. Grant, Scott A. Lesley, Marc-André Elsliger, Ashley M. Deacon, Ian A. Wilson

**Affiliations:** 1 Joint Center for Structural Genomics, La Jolla, California, United States of America; 2 Stanford Synchrotron Radiation Lightsource, SLAC National Accelerator Laboratory, Menlo Park, California, United States of America; 3 Department of Integrative Structural and Computational Biology, The Scripps Research Institute, La Jolla, California, United States of America; 4 Protein Production Facility, Novo Nordisk Foundation Center for Protein Research, Faculty of Health Sciences, University of Copenhagen, Copenhagen, Denmark; 5 Protein Sciences Department, Genomics Institute of the Novartis Research Foundation, San Diego, California, United States of America; University of Queensland, Australia

## Abstract

Kanadaptin is a nuclear protein of unknown function that is widely expressed in mammalian tissues. The crystal structure of the forkhead-associated (FHA) domain of human kanadaptin was determined to 1.6 Å resolution. The structure reveals an asymmetric dimer in which one monomer is complexed with a phosphopeptide mimic derived from a peptide segment from the N-terminus of a symmetry-related molecule as well as a sulfate bound to the structurally conserved phosphothreonine recognition cleft. This structure provides insights into the molecular recognition features utilized by this family of proteins and represents the first evidence that kanadaptin is likely involved in a phosphorylation-mediated signaling pathway. These results will be of use for designing experiments to further probe the function of kanadaptin.

## Introduction

Kanadaptin (kidney anion exchanger adaptor protein), also known as solute carrier family 4 anion exchanger member 1 adapter protein (SLC4A1AP), human lung cancer oncogene 3 protein (HLC-3) or NADAP, is widely expressed in almost all mammal tissues [1], [2], localizes to the cell nucleus and mitochondria [2], [3], and is part of a central proteome comprising 1,124 proteins that are ubiquitously and abundantly expressed in human cells [4]. Mouse kanadaptin was originally proposed to be an adaptor protein involved in targeting the Cl∶HCO3 exchanger kAE1 to the plasma membrane and, hence, implicated in inherited kidney disease [1] [n.b. the mouse protein in ref 1 (Uniprot O54716, 507 amino acids) represents a truncated version (∼240 amino-acids shorter at the N-terminus) of the full length protein (Uniprot E9PX68, 744 amino acids)]. Later studies indicated that kanadaptin does not interact with kAE1 in human cells [5], and its function remains to be elucidated.

Phosphorylation is a critical mechanism that mediates the assembly and disassembly of protein complexes in cellular signal transduction processes. FHA domains recognize phosphopeptides phosphorylated by serine/threonine kinases and serve as domain-mediated phospho-dependent regulators of protein assembly. They are commonly found in many regulatory eukaryotic proteins involved in a diverse range of processes, such as DNA-damage response, transcription and cell cycle control [6], [7]. FHA domains typically contain 80–100 amino acids that form a β-sandwich composed of 11 β-strands. Most FHA domains recognize phosphothreonine (pThr) with additional specificity provided by residues following the target pThr residue, particularly at the +3 position. The highly conserved pThr binding site, which is located at one end of the domain, is formed by inter-strand loops that present an Arg-Ser-Arg[Lys] triplet. This triplet is posed to interact with the phosphoryl group on the target threonine, thereby conferring specific recognition of pThr.

**Figure 1:**
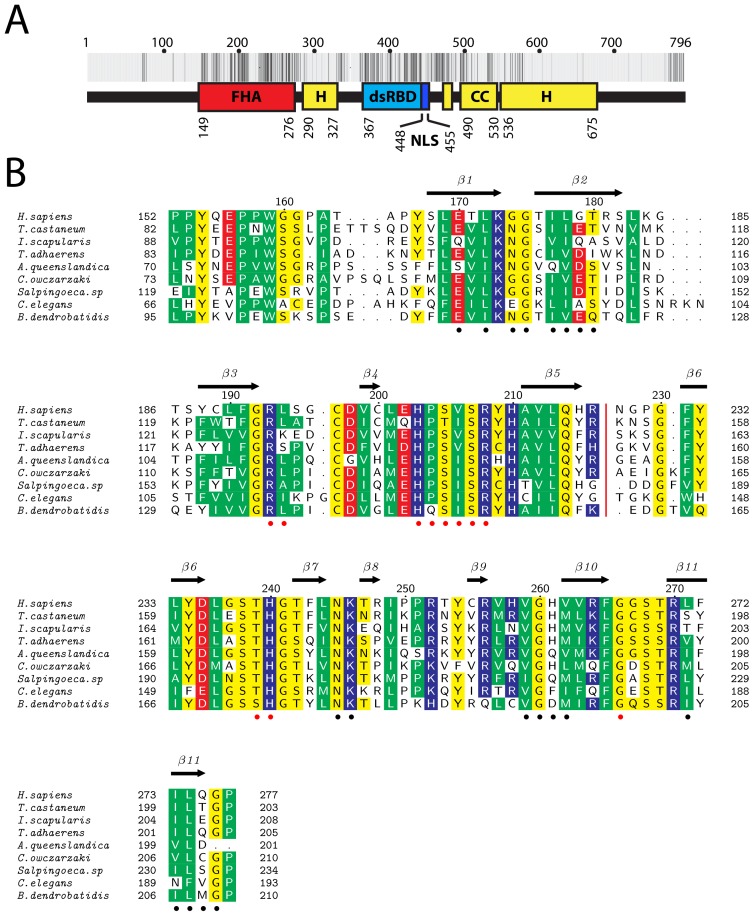
Domain architecture of the full-length human kanadaptin and multiple sequence alignment of the kanadaptin-FHA domains. (A) Domain architecture of human kanadaptin. FHA: fork-head associated domain, dsRBD: double-stranded RNA binding domain, H: helical region(s), CC: coiled-coil region, NLS: nuclear localization signal. Sequence conservation at each position of kanadaptin is represented by a vertical bar varying from non-conserved (white) to strictly conserved (black). (B) Multiple sequence alignment of representative FHA domains of kanadaptin orthologs. The secondary structure elements of human kanadaptin-FHA domain are shown on the top row. Residues involved in binding phosphopeptide or the dimeric interface are indicated by red or black dots respectively, at the bottom. Conserved residues are highlighted and colored according to their chemical properties (hydrophobic, green; polar and glycine, yellow; red, acidic; and blue, basic).

Sequence analysis indicates that the 796-amino-acids human kanadaptin contains at least two recognizable structured domains (Figure 1A): an FHA domain (residues 149–276) and a double-stranded RNA binding domain (residues 367–446 dsRBD). The nuclear localization signal (NLS) of kanadaptin is located immediately downstream from the dsRBD [3]. This domain architecture suggests that the nuclear protein kanadaptin might be involved in binding nucleic acids with its FHA domain serving as a regulatory module. Orthologs of kanadaptin are widely distributed in eukaryotes, from single-cell organisms such as *Capsaspora owczarzaki* and *Monosiga brevicollis*, to multicellular organisms such as *Caenorhabditis elegans* and humans, all of which contain a highly conserved FHA domain (Figure 1B). To gain insights into the function of human kanadaptin, we determined the crystal structure of its FHA domain at 1.6 Å resolution using the JCSG high-throughput structural biology pipeline [8] with protein expressed in the Protein Production Facility, Novo Nordisk Foundation Center for Protein Research, University of Copenhagen. The structure confirms the presence of a canonical pThr recognition site. Furthermore, a phosphopeptide mimic bound complex and a new dimer arrangement compared to other FHA dimers were observed in the crystal lattice, suggesting phosphopeptide binding dependent dimerization as a possible mechanism of kanadaptin activation.

## Results and Discussion

### Structure determination and the kanadaptin-FHA monomer

The FHA domain of kanadaptin was cloned and expressed in *Escherichia coli* with a TEV protease-cleavable expression and purification tag, and was purified by metal affinity and size exclusion chromatography. The purification tag was removed prior to crystallization, leaving two extra residues [Ser(−1) and Met(0)] not present in the native protein sequence. The FHA domain of kanadaptin was crystallized using the nanodroplet vapor diffusion method [20] with standard JCSG crystallization protocols [21] (see Methods). The structure was determined by molecular replacement in orthorhombic space group P2_1_2_1_2_1_ using the FHA domain of Pml1p subunit of the yeast precursor mRNA retention and splicing complex (PDB ID 3els) [9] as a phasing model, and refined to an R_cryst_ of 17.6% and an R_free_ of 19.8%. The final model has good geometry and compares favorably to other structures at similar resolution, with an overall MolProbity score [10] of 1.2 that ranks in the 99% percentile. All residues, except for one loop region (residues 222–227), are readily visible in the electron density map. The asymmetric unit (ASU) contains one homodimer (A and B), 207 water molecules, five glycerol molecules and six sulfate molecules. Glycerol and sulfate were present in the cryoprotectant and crystallization reagents, respectively. Data collection, processing and refinement statistics are shown in Table 1.

**Table 1:** Data collection and refinement statistics (PDB ID 4h87).

Data collection	
Space group	P2_1_2_1_2_1_
Unit cell	*a* = 34.8, *b* = 82.1, *c* = 82.5
Wavelength (Å)	0.97932
Resolution range (Å)	37.0-1.55
Highest resolution shell	1.63-1.55
No. observations	353,424
No. unique reflections	35,157
Completeness (%)^a^	99.9 (99.9)
Mean I/σ (I)^a^	27.4 (3.3)
R_merge_ on I^a^ (%)	4.8 (79.6)
R_meas_ on I^a^ (%)	5.1 (84.1)
R_pim_ on I^a^ (%)	1.6 (26.6)

Model and refinement statistics	
No. reflections (total)	35,081
No. reflections (test)	1,759
Cutoff criteria	|F|>0
R_cryst_ (%)	17.6
R_free_ (%)	19.8
Stereochemical parameters	
Restraints (RMSD observed)	
Bond lengths (Å)	0.010
Bond angles (°)	1.01
MolProbity score	
All atom clash score	2.5
Ramachandran plot (%)^b^	97.2 (0)
Rotamer outliers (%)	0.5
Average isotropic B-value (Å^2^)^c^	20.7 (26.7)
ESU based on R_free_ (Å)	0.079
No. protein residues/chains	248/2
Non-protein entities	207 H_2_O, 5 GOL and 6 SO_4_

a Highest resolution shell in parentheses.

b Percentage of residues in favored regions of Ramachandran plot (No. outliers in parenthesis).

c This value represents the total B that includes TLS and residual B components (Wilson B-value in parenthesis).

ESU = Estimated Standard Uncertainty in coordinates.

R_merge_ = Σ_hkl_Σ_i_|I_i_(hkl)-<I(hkl)>|/Σ_hkl_Σ_i_I_i_(hkl), R_meas_(redundancy-independent R_merge_) = Σ_hkl_[N_hkl_/(N_hkl_-1)]^1/2^Σ_i_|I_i_(hkl)-<I(hkl)>|/Σ_hkl_Σ_i_I_i_(hkl), and R_pim_(precision-indicating R_merge_) = Σ_hkl_[1/(N_hkl_-1)]^1/2^Σ_i_|I_i_(hkl)-<I(hkl)>|/Σ_hkl_Σ_i_I_i_(hkl).

R_cryst_ = Σ| |F_obs_|-|F_calc_| |/Σ|F_obs_|, where F_calc_ and F_obs_ are the calculated and observed structure factor amplitudes, respectively.

R_free_ = as for R_cryst_, but for 5.0% of the total reflections chosen at random and omitted from refinement.

**Figure 2:**
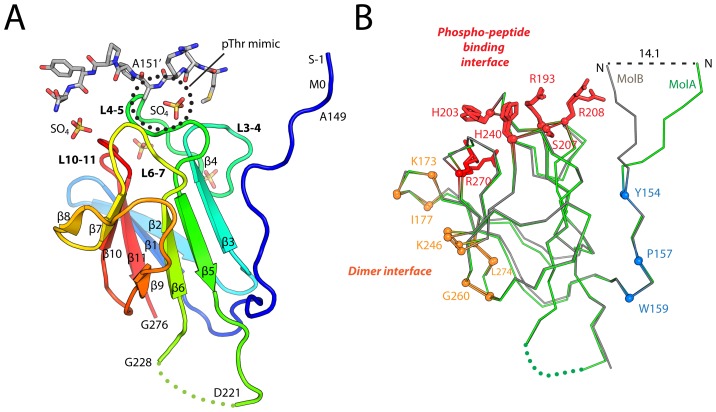
Structure of the FHA domain of kanadaptin. (A) Ribbon representation of the structure colored from N-terminus (blue) to C-terminus (red). Secondary structure elements; β-strands are labeled β1 to β11, and loops between consecutive β-strands (x and y) are labeled as Lx-y. Sulfate ions and the peptide segment from a crystallographic symmetry-related molecule are shown as sticks. (B) Structural comparison of the two kanadaptin-FHA molecules in the ASU (A: green and B: gray). Conserved residues are shown in ball-and-stick and colored by functional category (dimerization: orange, phosphopeptide-binding: red, and the N-terminal region: blue).

In common with other FHA domains, the FHA domain of kanadaptin adopts a β-sandwich fold consisting of 11 β-strands (antiparallel β-sheet1: β2, β1, β11, β10, β7, and β8; mixed β-sheet2: β4, β3, β5, β6, and β9; Figure 2A). The two monomers in the ASU are very similar [Figure 2B, RMSD of 0.5 Å for 117 Cα atoms between residues 154–276], except for the N-terminal region, which displays a 14 Å displacement between monomers. This large displacement is due to the N-terminus of molecule B binding the putative phosphopeptide binding site of monomer A of a symmetry-related dimer. Residues that are conserved among orthologs (Figure 1B) are clustered in the phosphopeptide binding site, the dimerization interface (Figure 2B), and also includes a few residues at the N-terminus (Tyr154, Pro157, and Trp159) that pack against β-sheet 2, thereby protecting it from solvent exposure.

### Phosphopeptide binding site and a mimic-bound complex

**Figure 3:**
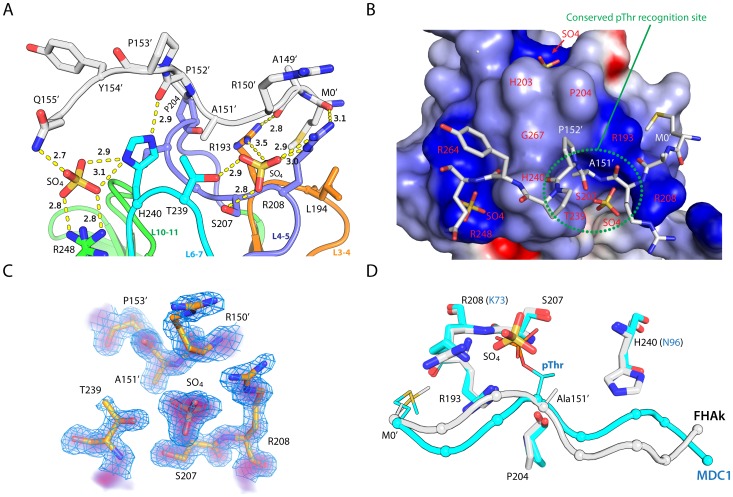
Recognition of a phosphopolypeptide mimic by the FHA domain of kanadaptin. (A) Interaction between the peptide (gray), the sulfates (orange) and the kanadaptin-FHA domain. Each loop involved in binding peptide and sulfate is in a different color. Hydrogen bonds are denoted by dashed lines, and corresponding distances in Å are indicated. Residues from the symmetry-related molecule are indicated by primed symbols. (B) Electrostatic surface potential of the kanadaptin-FHA domain (scale from −10 to +10 kT/e; blue, positive; red, negative). The bound peptide and sulfates are shown as sticks. (C) 2Fo-Fc density near the putative pThr binding site. The mesh (blue) is contoured at 1.0 sigma level, and the density level is represented by a linear color gradient from blue (1.0 sigma) to red (5.0 sigma). (D) Comparison of the ligand conformation in FHA domains of kanadaptin (gray) and MDC1 (cyan, PDB ID 3unn) with the peptide ligand represented as tubes with Cα atoms marked by spheres. Side chains of ligands [methionine and pThr (or Ala151/SO_4_ in kanadaptin] and receptors are shown as thin and thick sticks respectively.

The putative phosphopeptide binding site, formed by the loop connecting β-strands 3 and 4 (L3–4), L4–5, and L6–7 (Figure 3A), has positive electrostatic potential (Figure 3B). Interestingly, the N-terminus of a symmetry-related molecule (Met0-Ala149-Arg150-Ala151-Pro152-Pro153-Tyr154-Gln155, where Met0 is the N-terminal methionine from the expression construct) as well as a sulfate ion from the crystallization reagent are bound at the phosphopeptide binding site of monomer A (Figure 3A–B). The sulfate group (estimated occupancy ∼0.8, average B-value 20 Å^2^) and the peptide (average B-value 28 Å^2^) are both well-ordered with excellent electron density (Figure 3C); their average B-values are comparable to the protein (25 Å^2^). The backbone atoms of the bound N-terminal portion of the FHA forms multiple hydrogen bonds to Arg193, Arg208, and His240 of the pThr recognition site, while the sulfate group hydrogen bonds with Ser207, Thr239, Arg193, and Arg208 (Figure 3A). All these surface residues are strictly conserved among kanadaptin orthologs (Figure 1B) and thus indicative of a common pThr binding site. Met0, Ala151 and Pro152 also form van der Waals contacts with the protein. Together, the bound peptide and sulfate have a very similar arrangement to the pThr peptide in MDC1 [9]. Superposition of the first five equivalent Cα atoms of the peptides of the kanadaptin-FHA domain and MDC1 results in an RMSD of 1.3 Å (the distance between the two equivalent Cα atoms at the pThr site, Ala151 of the kanadaptin-FHA domain and pThr of MDC1, is 0.6 Å, Figure 3D). Therefore, the sulfate ion and the bound peptide, likely substitute for the pThr-containing peptide, with the sulfate corresponding to the phosphate of pThr.

In contrast, monomer B represents a ligand-free state, with its binding site occupied by waters. Nevertheless, the conformation of the phosphopeptide binding site is very similar to that of monomer A (Figure 2B), in agreement with the reported rigidity of these sites in other FHA structures [6]. Two additional, conserved sites near the putative pThr binding site are occupied by sulfate ions (Figure 3B). The first site is partially conserved and formed by His240, Arg248 and Arg264 (Figure 3A), while the second site is completely conserved and formed by Lys173 from L1–2, Ser268, Thr269 and Arg270 from L10–11, and Glu202 and His203 from L4–5. Notably, residues of the second site (Ser268, Arg270, and Lys173) are arranged in a similar fashion to the canonical pThr binding site residues (Ser207, Arg208 and Arg193). These additional binding sites could indicate an extended recognition surface for anionic groups of potential ligands. Recognition of more than one phosphorylation sites by an FHA domain, such as observed in the FHA domain of Dun1, can significantly increase the binding affinity [11]. The potential, second binding site of kanadaptin-FHA is located on the opposite side compared to Dun1-FHA, with respect to the common, conserved pThr-binding sites.

### Structure comparisons

**Figure 4:**
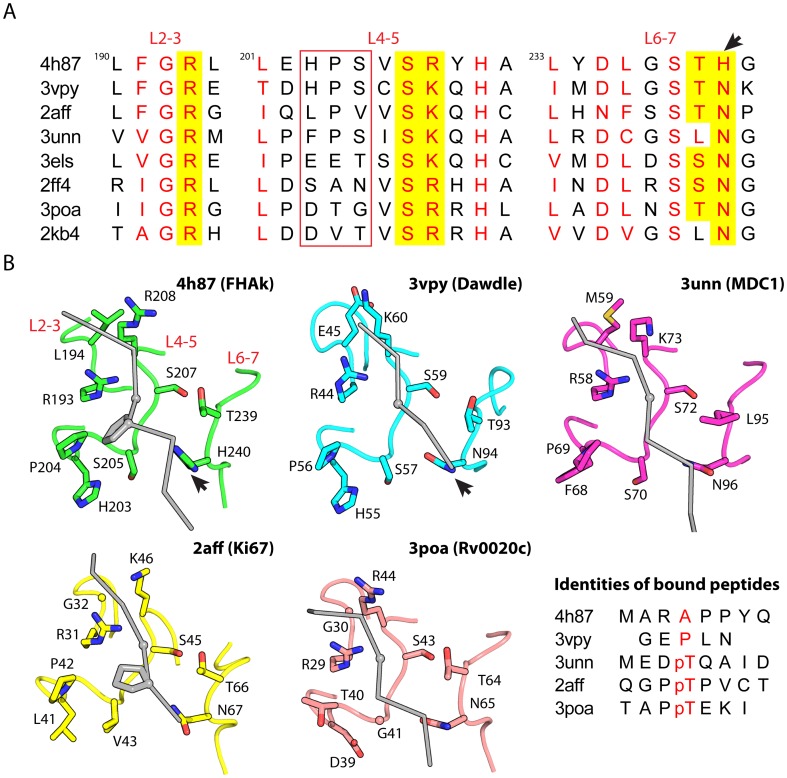
Comparison of the kanadaptin-FHA domain and other FHA structures. PDB ID's and corresponding protein identities are as follows, 4h87: the kanadaptin-FHA domain, 3vpy: Dawdle, 2aff: Ki67, 3unn: MDC1, 3els: Pml1p, 2ff4: EmbR, 3poa: Rv0020c, and 2kb4: OdhI. (A) Structure-based sequence alignment of the conserved loops involved in binding pThr-containing peptides. Conserved residues are colored in red, and residues that are directly involved in binding ligand are highlighted over a yellow background. The variable region in L4–5 in FHA domains listed above (but conserved in kanadaptin homologs) is marked by a red box. His240 and equivalent residues in other FHAs (Asn) are marked by arrows in (A) and (B). (B) Comparison of the pThr-containing peptide recognition sites, shown in similar orientations. Residues near the binding sites are shown as sticks. The bound phosphopeptides or mimics are shown as a gray tube (gray) with the location of the pThr Cα is shown as a sphere on the gray tube. Sequences of the bound peptides are shown in the bottom right corner.

The structure of the FHA domain of kanadaptin is very similar to that of other FHA domains. For example, it aligns with the *Arabidopsis thaliana* Dawdle FHA domain (PDB ID 3vpy) [12] with an RMSD of 1.8 Å over 120 Cα atom pairs and a sequence identity of 33%. The putative pThr recognition site is also very similar to that of other FHA domains [9], [13]–[16] (Figure 4), in particular the Dawdle FHA domain. One residue of particular interest in the pThr binding region is His240 that is strictly conserved in all FHA domains of kanadaptin orthologs (Figure 1B), but is not conserved across other non-kanadaptin FHA domains where Asn is instead found at the equivalent position (Figure 4A, marked by an arrow). However, in both kanadaptin FHA and non-kanadaptin FHA domains, the side chain at this position (Asn or His) hydrogen bonds with the backbone carbonyl group of the amino acid immediately following the pThr amino acid (pThr+1, in the case of the kanadaptin-FHA domain, Pro152). Therefore, this structurally conserved residue functions in anchoring the target peptide within the recognition site and may also help define the preferred residue at pThr+1 (e.g. a proline). This is consistent with other studies that have explored the side chain specificities of the pThr binding site. For example, it has previously been shown that peptide specificity is modulated by the chemical nature of the side chains at positions pThr+3, +1, −2 and −3 [6], [7]. Overall, peptides bound to FHA domains share a comparable conformation (Figure 4B). These structural similarities suggest that the bound polypeptide and sulfate ion are structurally and functionally relevant and provide the first structural insights into the molecular recognition motifs used in this human protein.

### The kanadaptin-FHA dimer

**Figure 5:**
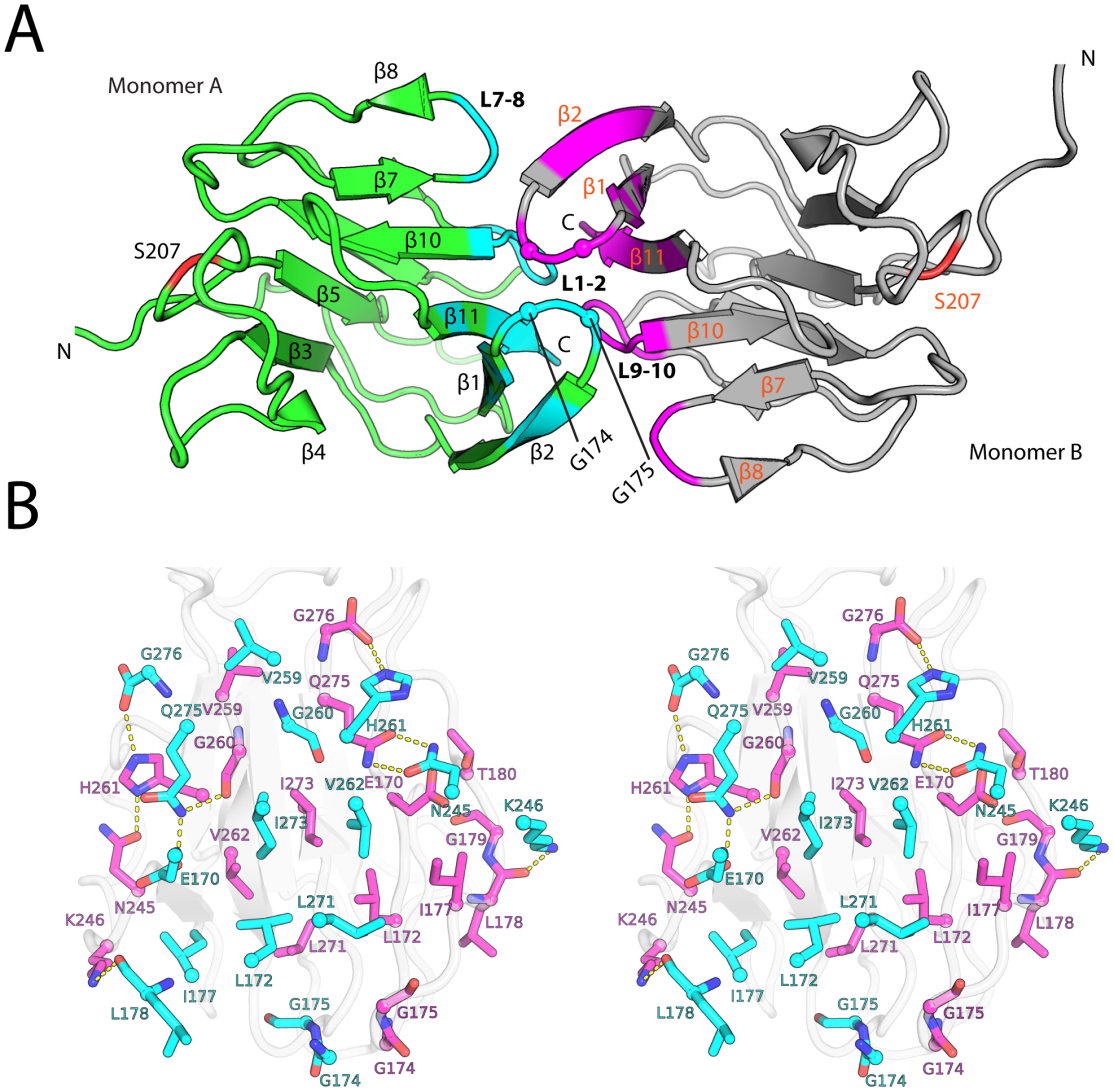
The kanadaptin-FHA dimer in the crystal asymmetric unit. (A) The kanadaptin-FHA dimer (molecule A: green, molecule B: magenta). Residues near the dimer interface are highlighted (cyan or purple). Ser207 located close to the canonical pThr recognition site is highlighted in red. Gly174 and Gly175 from L1–2 are shown as spheres. (B) Stereoview of the dimer interface (molecule A: cyan, molecule B: gray/magenta). Residues involved in the dimer interface are shown as sticks, and hydrogen bonds as yellow dashed lines.

A homodimer is identified in the crystal lattice based on analysis of contact interfaces. The kanadaptin-FHA domain dimerizes via residues on β-sheet1 of each monomer (Figure 5A), burying ∼751 Å^2^ of solvent accessible area per monomer. The two β-sheets of opposing monomers pack in a face-to-face arrangement with the phosphopeptide binding sites on the outer surfaces, distal from the dimerization interface. The dyad axis is approximately parallel to the β-strands. The last β-strand is buried in the dimer interface. The dimerization interface involves a core set of hydrophobic residues in the center of β-sheet1 (Leu172, I177, Val259, Gly260, Val262, Leu271, and Ile273), and additional residues at the perimeter involved in backbone hydrogen bonding (Leu178, Asn245, Lys246, His261, Gln275, and Gly276; Figure 5B). Dimerization interface residues are contributed from L1–2, L7–8, L9–10, β1, β2, and β11. In particular, Gly174 and Gly175 from the L1–2 loop facilitate packing of adjacent loops from neighboring molecule (Figure 5A). The dimer of the kanadaptin-FHA domain differs from other FHA dimers, such as MDC1-FHA [9] or Chfr-FHA [17].

Analytical size exclusion indicates that the FHA domain of kanadaptin exists as a monomer in solution (data not shown). Thus, the physiological relevance of the kanadaptin-FHA dimer observed in the crystal is currently unclear. However, we postulate that such a dimer may mimic a phosphopeptide-bound state (see below), and could possibly represent a physiologically relevant state (e.g. activated). Indeed, phosphopeptide-mediated FHA dimerization appears to be a common strategy utilized by many FHA-regulated signaling pathways [6]. In several well-studied cases, the FHA domain binds phosphopeptides harbored in another region of the same protein [6], for example, at the N-terminus for MDC1 [9], which is analogous to the inter-chain (self) recognition that we observe within the kanadaptin-FHA homodimer structure.

### Functional implications

The ubiquity of kanadaptin in mammals suggests that it should have an important physiological function. The structure of the kanadaptin-FHA domain supports current hypotheses that kanadaptin participates in cell signaling pathways via its FHA domain. FHA-containing proteins generally possess one or more “functional” modules, whose activity is regulated by phosphopeptide binding. In kanadaptin, the “functional” module is potentially the predicted dsRBD (Figure 1A), which shares significant sequence similarity to other dsRBDs (e.g. dsRBD1 of human RNA helicase A, sequence id 23% [18]). dsRBDs are common modules that play critical roles in nucleic acid binding in diverse cellular functions [19]. Indeed, homology modeling suggested that dsRBD also contains a conserved positively charged surface (data not shown), consistent with the potential to interact with nucleic acids.

Secondary structure predictions indicate full-length kanadaptin contains two helical regions between the FHA domain and the dsRBD domain (residues 290–327), and after the dsRBD domain (residues 470–675, Figure 1A). In addition, a coiled-coil region is predicted towards the start of the second helical region (residues 490–530). The arrangement of the C-terminal portions of the FHA dimer suggests that the helical (or coiled-coil) region connecting the FHA domain and the dsRBD domain may also interact upon dimerization of the FHA domains in the full-length kanadaptin. Therefore, we propose that the RNA-binding activity of the dsRBD domain of kanadaptin may be regulated by the oligomeric state of the FHA domain, which in turn is controlled by binding of a phosphopeptide.

We propose that the putative interaction with a pThr-peptide by kanadaptin is similar to the interaction observed with the peptide and the sulfate in the crystal structure. Further experiments such as phosphopeptide library screening, pull-down assay and site-directed mutagenesis may shed light on the identity of potential binding partners, and ultimately the physiological role of kanadaptin. The structure presented here provides a structural framework for further investigations into the cellular function of kanadaptin.

## Materials and Methods

### Cloning

Clones were generated using Ligation Independent Cloning (LIC). The gene encoding the FHA domain of kanadaptin (UniProt: Q9BWU0 or NADAP_HUMAN, residues 149–276) was amplified by polymerase chain reaction (PCR) from the Invitrogen Ultimate collection using Phusion DNA polymerase (NEB) and forward primer, 5′-tacttccaatccatgGCCCGGGCTCCCCCC-3′ and reverse primer, 5′-tatccacctttactgttaTCCCTGCAGGATAAAGAGCCGGG-3′ (target sequence in upper case). The resulting DNA was inserted into the expression vector pNIC28-Bsa4 using LIC. The expression vector encodes an amino-terminal tobacco etch virus (TEV) protease-cleavable expression and purification tag (MHHHHHHSSGVDLGTENLYFQ/S). The DNA insert and the vector were both prepared for LIC by treatment with restriction enzyme digestion and T4 DNA polymerase. *Escherichia coli* MachI (Invitrogen) competent cells were transformed with the treated DNA insert and vector and dispensed on to selective LB-agar plates. The success of cloning was confirmed by DNA sequencing.

### Protein production

Protein expression was carried out using *E. coli* expression strain BL21 Rosetta2 (DE3) R3 T1. 50 ml of TB media containing 50 µg/ml kanamycin and 25 µg/ml chloramphenicol was inoculated with cells from a glycerol stock. The overnight culture was grown at 37°C and used the following morning to inoculate 4.5 L of TB media containing 50 µg/ml kanamycin. The expression culture was grown at 37°C to an OD_600_ = 1.65. The temperature was then reduced to 18°C, and expression induced by adding IPTG to a final concentration of 0.5 mM. The cells were harvested 19 hours after induction by centrifugation at 4000× g for 10 minutes.

The cell pellets were resuspended in lysis buffer (300 mM NaCl, 0.5 mM TCEP, 10% Glycerol, 100 mM HEPES pH 7.5) supplemented with Complete Inhibitor cocktail (EDTA Free) and Benzonase (750 U/100 ml) and the cells were lysed by three passes through a high pressure homogenizer at 1000 Bar (D20 Avestin). The lysate was centrifuged at 18500× g for 40 minutes and the supernatant filtered through a 0.22 µm PES filter. The filtrate was collected for purification. The proteins were initially purified using a two-step affinity and size exclusion chromatography using an ÄKTAxpress system (GE Healthcare). The affinity chromatography column (1 ml HiTrap Chelating) was equilibrated in binding buffer (300 mM NaCl, 0.5 mM TCEP, 10% Glycerol, 10 mM Imidazole, 20 mM HEPES pH 7.5) and the sample loaded onto the column. The column was washed (300 mM NaCl, 0.5 mM TCEP, 10% Glycerol, 30 mM Imidazole, 20 mM HEPES pH 7.5). The protein was eluted using a step gradient of elution buffer (300 mM NaCl, 0.5 mM TCEP, 10% Glycerol, 500 mM Imidazole, 20 mM HEPES pH 7.5) and fractions collected for further purification. A second purification step was carried out using a Superdex 75 PG 16/60 column pre-equilibrated with running buffer (150 mM NaCl, 0.5 mM TCEP, 10% Glycerol, 20 mM HEPES pH 7.5). Fractions were collected and the purification tag was cleaved off by overnight incubation with TEV protease (1∶100 molar ratio) at 4°C. The cleaved purification tag and the protein were separated by an additional pass over the affinity column. The protein was buffer exchanged into the final crystallization buffer (150 mM NaCl, 30 mM Imidazole, 0.5 mM TCEP, 20 mM Tris pH 8.0) using a PD-10 column (GE Healthcare) and finally concentrated to 8.0 mg/ml for crystallization trials. The identity of the protein was confirmed by electrospray ionization mass spectrometry (ESI-MS) of the intact protein.

### Crystallization

The FHA domain of kanadaptin was crystallized using the nanodroplet vapor diffusion method [20] with standard JCSG crystallization protocols [21]. Sitting drops composed of 100 nl protein solution mixed with 100 nl crystallization solution in a sitting drop format were equilibrated against a 50 µl reservoir at 277 K for 15 days prior to harvest. The crystallization reagent consisted of 1.6 M ammonium sulfate and 0.1 M citric acid pH 5.0. Glycerol was added to a final concentration of 20% (v/v) as a cryo-protectant. Initial screening for diffraction was carried out using the Stanford Automated Mounting system (SAM) [22] at the Stanford Synchrotron Radiation Lightsource (SSRL, Menlo Park, CA). The diffraction data were indexed in orthorhombic space group P2_1_2_1_2_1_.

### Data collection, structure solution, and refinement

Native data were collected at wavelength 0.97932 Å at 100 K using a Pilatus 6M detector (DECTRIS) at SSRL beamline BL11-1. The data were processed by an automation script [23] that runs XDS [24]. The structure of the FHA domain of kanadaptin was determined by molecular replacement (MR). Initial MR “hybrid” model templates were created [25] using the phenix.mr_model_preparation tool [26], which removes poorly aligned regions and trims side-chain atoms of non-conserved residues based on sequence alignments between the target sequence and top homologs in PDB calculated with the HHpred server [27]. Multiple molecular replacement trials were carried out in parallel on a computer cluster with each job exploring different combinations of parameters (models, resolution, model completeness, and sequence similarity). Each job includes an MR step implemented in MOLREP [28], a rigid-body and restrained refinement step in REFMAC5 [29], followed by automatic model rebuilding in ARP/wARP [30]. A MR solution was identified from a trial using the FHA domain of the Pml1p subunit of the yeast precursor mRNA retention and splicing complex (PDB ID 3els) [15] as the search model. The resulting ARP/wARP model had an R_cryst_ of ∼20% and good completeness, and was confirmed by manual inspection of the corresponding density maps. Further model completion and refinement were performed manually with COOT [31] and BUSTER [32]. The refinement included TLS refinement with one TLS group per monomer and NCS restraints. Data and refinement statistics are summarized in Table 1. Analysis of the stereochemical quality of the model was accomplished using MolProbity [10]. Molecular graphics were prepared with PyMOL (http://www.pymol.org/). Electrostatic potentials were calculated using the program Delphi [33]. The structure factors and atomic coordinates are deposited in the RCSB Protein Data Bank (http://www.rcsb.org) with PDB codes 4h87.

### Sequence analysis and alignment

Identification of domains and definition of domain boundaries were carried out using PFAM [34] and HHpred [27]. Secondary structure prediction was carried out using PSIPRED [35]. Coiled-coil regions were predicted using MARCOIL [36] and COILS/PCOILS [37]. Homology modeling was performed with MODELLER [38] and I-TASSER [39]. Sequence alignments were calculated with CLUSTAL W2 [40], and rendered using TeXshade [41].
